# “Your Ovaries Are Expired, Like an Old Lady” Metaphor Analysis of Saudi Arabian Women’s Descriptions of Breast Cancer: A Qualitative Study

**DOI:** 10.3389/fpsyg.2022.924934

**Published:** 2022-07-22

**Authors:** Wafa Hamad Almegewly, Maha Hamed Alsoraihi

**Affiliations:** ^1^Department of Community Health Nursing, College of Nursing, Princess Nourah bint Abdulrahman University, Riyadh, Saudi Arabia; ^2^Department of Applied Linguistics, College of Languages, Princess Nourah bint Abdulrahman University, Riyadh, Saudi Arabia

**Keywords:** breast cancer, experience, metaphors, Saudi Arabia, patients

## Abstract

**Background:**

Assessing and understanding the language that women use to express physical, emotional, and social concerns of breast cancer experiences can often be overlooked, even though there is evidence that effective communication between cancer patients and health care providers improves quality of life. This study aims to assess the use of metaphors in conceptualizing breast cancer experience lived by Saudi Arabian women.

**Materials and Methods:**

This is an interpretative phenomenological qualitative study, a purposeful sample of 18 breast cancer patients at an oncology outpatient’s clinic in Saudi Arabia were invited to engage in face-to-face interviews. Data was analyzed using Metaphor Identification Procedure (MIP).

**Results:**

Four themes were constructed: dark hidden force, battling imminent death, dreaming and awakening calls, and inner and outer transformation.

**Conclusion:**

Identifying metaphors may be beneficial toward improving communication between health care providers and breast cancer patients, who often experience difficulties expressing their needs.

## Introduction

Breast cancer is the most common of cancer and leading cause of death among Saudi women and accounted for 29% of all newly diagnosed cancer among them (The World Health Organization, 2020a). Moreover, it is estimated that by year 2040, the annual incidence will reach to 6,886 (The World Health Organization, 2020b). Compared to other countries with mean age of (63), breast cancer is commonly a reported problem among Saudi women in younger age of 45 (Asiri et al., 2020). Breast cancer patients experienced physical symptoms associated with treatment side effects as fatigue, insomnia, cognitive and sexual dysfunction including libido, infertility and weight gain. In terms of sociopsychological problems, they reported signs of stress, uncertainty, fear of cancer recurrence, and depression. Breast cancer patients globally may share similar experiences with treatment side effects but differing cultural values could influence how women perceive their illness experience. Previous research in Saudi Arabia suggests that women hold unique views on cancer as a deadly disease, including having to live hidden as a breast cancer patient; the role of spirituality and faith in coping; and family social support (ALmegewly et al., 2019). Some of these unique views are expressed in metaphor.

Metaphor is more than simply words or phrases; it is a cognitive method of communication embodied in our daily experiences (Potts and Semino, 2019). The use of metaphors and analogies has been commonly used in health and illness communication, specifically in the context of cancer discourse. Cancer patients often used metaphors that illustrate how they talk and think about cancer and make sense of their personal experience. Metaphors often clearly reflect both human suffering and sociocultural perspectives. Using metaphors helps in developing common way of language can be used and understood by both patients and healthcare providers. Also, it can fill the gap between the personal conceptual meanings of breast cancer experience and its medical framework. The study of metaphors as manifestations of human cognition has played a vital role in the fields of psychology, philosophy, linguistics and other disciplines over the past few decades. It is also employed as an analytic method, Metaphor Identification Procedure (MIP) (Group, 2007), as in this study.

Usually, people use language to communicate their intended messages and build shared common grounds with others. However, using metaphors as a specific tool of language is far more than just adding meaning to their thoughts; metaphors are used to communicate relations and build up personal identities (Gee, 2014). There are basically two important perspectives on how metaphors are applied by either speakers or writers. These two aspects are the rhetorical and cognitive perspectives. The rhetorical one perceives metaphors as “thoughtful and deliberate constructions,” i.e., the “poetic use of metaphors (Cameron, 2018). On the other hand, Lakoff and Johnson adopted the cognitive perspective in their publication “Metaphors We Live By” in 1983, in which they pointed out how metaphors are crucial to people’s ways of thinking and acting in everyday life (Lawler et al., 1983). They even went further to perceive people’s entire conceptual system as “fundamentally metaphorical in nature.” In other world, the use of metaphors reflects people’s awareness of the world around them and their understanding of their thoughts and actions.

Breast cancer patients are overwhelmed with many stressors during their diagnosis, treatment and beyond, which may not often verbally express. Investigating metaphors that women use in their descriptions about living with breast cancer can help to gain better insight into their thoughts, feelings, and attitudes about their disease. An individual’s ability in shaping meaning to her illness may reflect on her coping abilities during the illness, as well as during her overall recovery (Lipowski, 1970). Using positive expressions by breast cancer patient and her partner reflect good adjustment with the disease unlike families who used negative emotional words that indicate poor coping, stress and conflict within the family dynamic (Robbins et al., 2014). Furthermore, a recent study conducts five experiments to examine the effect of using particular metaphors on evaluating emotional and difficult situations such as cancer and other types of illness. In other words, this study aims at answering the question: “Can metaphors also influence the way we evaluate, or appraise, an emotionally distressing situation like an illness” (Hendricks et al., 2018). It was found that using different metaphors such “battle” or “journey” can affect patients’ reaction and evaluations of their hardship situations. According to analysis of patients and health care providers written online materials, it showed that neither the *“battle”* nor the *“journey”* were intrinsically harmful or good for everyone (Semino et al., 2017). This indicated that health care providers should be careful in using such these metaphors expressions with their patients. Reported that the violence metaphors associated with caner should not be heavily utilized by health care providers because it may have some depowering meanings. They instead suggest that health care providers should encourage their patients to use individualized metaphors that fit their experience (Demjén and Semino, 2020).

Some psychological reactions to cancer were reported by some patients in their narratives including, concealing their symptoms from their families and health care providers, in order not to be burden on others including clinicians (Byrne et al., 2002). Thus, the latter study and the current research, in turn, may inform medical practitioners about effective methods or measures of clinical communication, assessment and coping. As may cancer patients showed understanding while communicating with oncologist if the latter used metaphors in their talk (Casarett et al., 2010).

This study believes that the analysis of metaphors in the speech of breast cancer survivors will help in providing a deeper understanding of how these patients conceptualize and communicate about their experience in the process of dealing with and healing of their illness. Metaphors such as “fighting the disease,” “attack my body,” “win/lose the battle” have been used constantly by breast cancer patients. Many studies used heterogeneous group of patients at different stages of treatment (Byrne et al., 2002) and few focused on young breast cancer patients. Also, using qualitative design, and interviews provide more patient- oriented perspectives than quantitative studies. Excessive linguistic research has been tackled the cancer context in English, Spanish and other European languages (Khan et al., 2012; Appleton and Flynn, 2014; Cheung and Delfabbro, 2016; Magaña, 2020). However, few studies have been done in Arabic language, especially in medical contexts. Also, the reasons behind the use of such metaphors in such contexts especially from the Arabic language still needs to be discussed and thematically analyzed in ways that will help in developing a greater understanding of this journey by both patients themselves and health care providers. There was only one study that examined data was mainly some online narratives of breast cancer patients where metaphors were detected using corpus linguistics to find out “which metaphors are more frequent in the construction of cancer experience” (Abaalalaa and Ibrahim, 2022). This type of data analysis is limited to the metaphorical lexical units found in the patients’ written narratives without examining the non-linguistic factors that have impacts on directing the data discussion such as facial expressions, emotional and physical reactions which can be traced in conducting face to face interviews. Thus, this study focuses on the use of conceptual metaphors by interviewing Saudi breast cancer patients in their spoken narratives and investigates their linguistic expressions together with detecting their emotional and physical reactions which eventually validate their usage of such metaphors to depict vividly the whole experience.

This study aims to contribute to that gap in the literature by examining the use of metaphor in in-depth interviews conducted with Saudi Arabian breast cancer patients, guided by the following research question: *How do Saudi Arabian women express their experiences with breast cancer using metaphors?*

### Literature Review: Metaphors and Breast Cancer in Cultural Context

#### Metaphor and Linguistics

Metaphors can be identified as a figurative expression that can be used to substitute other linguistic expressions. They facilitate the process to “understand one thing in terms of another” (Lawler et al., 1983). Metaphors have been seen as ways of manifestations of human cognitive thoughts (Lawler et al., 1983). Al-Hasnawi (2007) investigated translated conceptual metaphors in religious texts and he concluded that the cognitive technique is preferred to approach translated metaphors. This is because identifying metaphors should be based on a socio-cultural understanding of both the source and the target languages. This also supports the claim that people are defined as culturally human beings where their stories can be discovered through the deep understanding of the meaning of metaphors.

Lawler et al. (1983) presented several examples in which they explained how metaphors can be used by ordinary people in everyday contexts where they differentiate between the concrete and abstract levels of conceptual metaphors. They emphasized that metaphors can be understood as a way of describing one conceptual framework in terms of another. They described two main domains where the source domain is usually the concrete level, and the second one is the target domain which is the abstract level and can be understood by referring to the concrete one (Kövecses, 2010). Conceptual metaphors can be found in some linguistic expressions that can be used in everyday conversations where few people can recognize them as metaphoric expressions. This realization forms the basis of Steen et al. (2010) claim where emphasis is placed on the assumption that metaphors can be realized in three- dimensions: pervasive, thought, and communication. It is the communication dimension which manifested how people can use metaphoric expressions in their daily conversations without realizing the fact that they are metaphoric expressions. This argument proves that metaphors can be embedded in the language where can be used deliberately or non-deliberately in some cases (Steen et al., 2010). This proves that the theory of Lakoff and Johnson of metaphors can be realized as valid and accepted.

#### Breast Cancer and Metaphor

An extensive body of literature on illness discourse such as dementia (Castaño, 2020), Alzheimer’s disease (Zimmermann, 2017), diabetes (Youngson et al., 2015), infertility (Palmer-Wackerly and Krieger, 2015), analyzed the use of metaphors in how patients openly disclose and evaluate their emotions and attitudes. In the literature, examining cancer discourse has been extensively discussed as it found that cancer patients tend to use metaphors in certain pattern. Different metaphors were widely examined in the context of violence such as, “battle,” “war,” “fight,” and “survivor” by both cancer patients, health care providers, family and friends (Hommerberg et al., 2020; Magaña, 2020). It is evident that many cancer patients used the latter violence metaphors because of the sense of control or empowerment that they construct and feel. Literatures often discuss the breast cancer as battles, ways of cure and other aspects. Only health care workers know that the truth is not as simple as readers perceive. Despite the prevalence of using the metaphor of “survivor,” many cancer patients argued that the latter term do not necessarily represent their identity (Rees, 2018; Lundquist et al., 2020). The truth is what behind the diagnosis and management of cancer and the process patients go through during and after diagnosis. In conclusion there are no perforable metaphors across languages or cultures that best describe cancer because each person has different cancer experience and expectation.

Structural metaphors such as, “scary,” “shocking,” and “fearful” are largely used by female cancer patients including breast by (66.67%) compared to males (33.33%) (Pei and Collin, 2020). Some metaphors linked to body image changes among this, losing breast and hair loss. Some metaphors used to describe the way that the tumor grow and then spread and aggressively attack other organs (Magaña, 2020). Describing cancer experience as a “journey” is another metaphor used among cancer patients, because it does not involve any sense of winning or losing as war or any other violence metaphors (Pei and Collin, 2020).

Furthermore, a recent study focuses on the themes of violence and resistance in using metaphorical language by cancer patients. It investigated the metaphorical expressions of violence in the responding language of cancer patients and how these metaphors are perceived in health domains. Through a detailed analysis of six case studies, it aimed at showing how such combination of both metaphorical detection and violent framework “constitutes a useful and structured method for understanding the nature of resistance standpoints and the specific dimension (s) of metaphor they address” (Wackers et al., 2021). Another similar and recent study focuses also on theme of fighting and resisting found on the use of metaphors in the language of cancer patients. It concluded in finding out the value of using such metaphors by those incurable breast cancer patients to share their experience in “a supportive context and to reinvest the war metaphors in order to express themselves in a more authentic way” (Guité-Verret and Vachon, 2021).

## Methodology

### Research Design

This is an interpretative phenomenological qualitative study which is based on understanding the human lived experience within the cultural, social, and religious perspectives (Heidegger, 1927). The metaphors in this research are examined within the framework discourse analysis. In its basic definition, discourse analysis focuses on “language -in-use” and how it is used “to say things, do things, and be things” (Gee, 2014). Language is used to accomplish seven “building tasks”: significance, identities, activities, relationships, connections and sign systems, politics, and knowledge (Gee, 2014). Moreover, metaphors go side by side with other three features of discourse analysis described by the latter author: situated meaning, intertextual, and figured world. These fundamentals of discourse analysis make metaphors an appropriate methodological choice for data analysis of this study.

### Participants and Setting

The metaphorical expressions were drawn from interviews with Saudi Arabian breast cancer patients in a larger qualitative study. A purposive sample of 18 breast cancer patients were recruited from an oncology outpatient clinic in one selected hospital in a Saudi Arabian city. The health care center is one of the largest and advance cancer center in the middle east region that provides primary, secondary and tertiary care. These women were primarily diagnosed with breast cancer, they aged between 30 and 50 years, in a premenopausal status, finished treatment 6–47 months before interviews took place, and with no current history of mental illness or advance cancer stage (metastasis) and Arabic speakers. The inclusion criteria were set to maintain the homogeneity of the participants in terms of age, cancer stage, and cultural background. The eligible participants were invited by the health care providers working in the oncology out-patient clinic at the selected hospital.

### Data Collection Measurement

Semi-structured in-person interviews, guided by relevant literature, were conducted to inquire into the women’s descriptions of their experiences of being breast cancer patients. The interview questions were created in a chronological order starting with women’s experience with breast cancer diagnosis, treatment, and finally survivorship. The questions were: “could you kindly tell me about your breast cancer journey, starting with how you were diagnosed?”- “what did you feel when you start your treatment?”- “how was it for you and your family members?” and “to what extend have breast cancer impact your life?”

This study is trying to bridge the gap between the use of metaphors in public discourse and its use by individual narrating personal health issues. This study shows the joining efforts of two academic scholars whose major happen to reach mutual interests in carrying out this project. Data of this study has been gathered by the first author, there were face to face interviews, in Arabic language. The interviews were conducted in a quiet private meeting room in the selected hospital. All the interviews were digitally recorded and then verbatim transcribed by the first author. Some research participants were opened in sharing their experiences with the first author, because she showed them her understanding as she is coming from similar cultural background and speaking the same language. The first author did the translation of texts from its original language (Arabic) into English and the second author whose major is Linguistics and bilingual, revised the translated data and check its accuracy and took it to other levels of metaphor analysis, aiming at exploring the mutual themes that can connect these individuals’, experiences together.

### Ethical Considerations

Ethical approval was granted from the selected hospital. Women’s involvement in the study was entirely voluntary and their consent was obtained prior to the interview. Also, participation information sheet was given to all the interested participants explaining the study aim which is understanding the participants’ experiences with breast cancer from the metaphors they used. Details such as names of the participants, location and contact numbers were kept anonymous throughout the study and within the findings. The participants were given names other than their real names (pseudonym), and their identifying information was safely locked away and digitally protected by passwords.

### Data Analysis and Rigor

This study reports upon analysis of metaphorical expressions used by Saudi Arabian breast cancer patients using MIP (Group, 2007), as it considered widely effective in metaphor analysis in a communicative discourse. MIP is used throughout the current study to identify the metaphorical expressions expressed by breast cancer patients. The linguistic analysis in this study is limited only to metaphors connected to breast cancer and, hence, they are identified within this limited context where participants used metaphors to reflect their experiences in facing this life-threatening disease.

MIP can be understood as the process of determining the relationship between a lexical unit and its possible metaphorical use in a particular discourse. Steps of MIP method can be summarized as the following:

Step. 1. Researchers of this study have to read thoroughly the collected data with the purpose of finding out the general meaning of the participants’ narratives.
Step. 2. Researchers need to determine the lexical units and find out whether such units have basic meanings or contextual connotations.
Step. 3. If lexical units have only basic meanings, then such units are marked as not metaphoric. If lexical units show contextual meanings that can be contrasted with the basic meanings, then such units can be marked as metaphoric (Group, 2007).

Firstly, the first author provided the raw data including the recorded interviews, and transcripts to the second author, a linguist to begin analysis using MIP. Secondly, metaphorical lexical units were identified, and meanings were determined within their contexts. Every word was examined as one lexical unit for metaphor identification purposes. Moreover, when a compound or a phrasal verb is identified, it would be coded as a single example of a metaphor (Cameron, 2018). Thirdly, meanings of these metaphorical expressions were deeply analyzed in relation to whether these are basic meanings or conceptual ones that could be compared to and identified. Finally, a linguistic unit can be considered as a metaphorical expression when it manifests both a basic meaning and a conceptual one within its context that can be compared to another one and allow for further analysis. A set of themes were developed followed consultations by first and second authors.

In order to increase the rigor of the results, principles of trustworthiness of qualitative research like credibility, conformability, translatability, and dependability were considered (Lincoln et al., 1985). Reflective notes were taken by the first author during and after interviews that include any important points, facial expressions, author’s feelings and any issues need to be covered in the next interviews. The multidisciplinary collaboration between the first author, who is specialized in nursing and the second author, who is coming from a linguistic background, has enhanced prolong engagement with the data, and increased the rigor of the results. This integration included sharing knowledge, experience, and identify emerging themes. Doble translation was done by the first and second authors to check the accuracy of translated text. Also, a number of verbatim quotes were used to support participants’ experiences and feelings.

## Results

The data reported by18 young breast cancer patients and their details are shown below in Table 1.

**TABLE 1:** Demographical profile.

Demographical profile	*N*	%
**Age**		
30–35	2	11
36–41	7	39
42–47	6	33
48–53	3	17
**Employment status**		
Unemployed	12	67
Employed	6	33
**Education**		
Primary	1	6
Secondary	4	22
University	13	72
**Family history of breast cancer**		
Yes	7	39
No	11	61

A rich array of metaphorical expressions clustered into four themes as shown in Table 2.

**TABLE 2:** Metaphor themes.

Metaphor themes	Examples
Dark hidden force	*Amar: “I’m afraid of the devil’s eye.” “I’m scared of the thing*…*you know.” Leena: “This disease put a curse on me (the evil’s eye).” Sahara: “Cancer is devil’s eye/magic.” Shatha: “Evil was sucked out.” Lama: “Surviving is like get rid of this thing.” Anoud: “Cancer looked like a demon was trying to kill me.”*
Battling imminent death	*Amar: “The phantom of cancer is still present.” Azizah: “The phantom of death is still here.” Aisha: “It was my end.” Aisha: “Swimming from death.” Shahad: “I felt I was drowning.” Shatha: “I was dying from inside.” Shatha: “I felt like I was going to die tomorrow.” Leena: “The woman is on a fight with a cancer to live, to survive.”*
Dreaming and awakening calls	*Asera: “My life is filled with pain and tears.” Aisha: “Minutes of waiting are killing me.” “A bad dream*…*then you woke up.” Alaa: “Like a flashback that would never stop.” Aisha: “It is a wakeup call.” Ohoud: “This disease was like a wakeup call.” Anoud:” This disease was a loving message.” Sahara: “This disease was a blessing/gifts from God and not a punishment.” Haia: “This disease is like a test from God.” Aisha: “Minutes of waiting are killing me.” “A bad dream*…*then you woke up.” Alaa: “Like a flashback that would never stop.” Sara: “This disease is like a break that let me think about my life.” Sara: “This disease is a test from God to see how patient I am.”*
Inner and outer transformation	*Asera: “His reaction has been shutting me down.” Asma: “I felt I was expired or useless person to him.” Asma: “your ovaries are expired*… *shut down just like an old lady.” Ohoud: “I was a patient; it is a label stick to me forever.” Shatha: “My feelings were up and down like a lift.” Asma: “I felt I was born again.” Haia: “I felt like a rebirth.” Asma: “broken from inside.” Sara: “I’m a defendable person and there is no room for weakness in my life.”*

These themes above reflect Saudi women’s experiences with breast cancer as covering a complex range of emotions—sometimes devastating, sometimes instrumental, sometimes inspiring—as illustrated in the sections below.

### Dark Hidden Force

Participants portrayed breast cancer as an “evil” that made participants feel afraid and too weak to control it. Shatha described: “The evil was sucked out after the surgery,” and Nourah said: “I thought if that thing [cancer] found its way into my body, it would spread outside my breast.” Amar and Anoud referred to the cancer as a phantom and demon, respectively: “The phantom of cancer is still present” (Amar) and “Cancer looked like a demon was trying to kill me” (Anoud). Many of the women never referred to breast cancer by its name, perhaps due to their fear of it; instead, they used different phrases like “the disease,” “the tumor,” and “the thing.” For example, Amar said: “I’m scared of the thing…you know.”

The dark force of breast cancer has a powerful effect on women’s life. Aisha described her life after a breast cancer diagnosis as “my life filled with darkness” which indicates an inability to see any light or hope at the end of her road with cancer. Leena said, “I was like a hypnotized person, saying ‘Yes’ to anything,” which is an interesting metaphor, indicating her lack of authority over cancer that takes over all her senses. Amar mentioned how lonely she was and Shatha described her painful experience with chemotherapy, claiming: “if someone had died in front of me, I would walk on and never look back.” This indicated a deep loss of sense, which made her unable to think beyond her own suffering experience. Her condition was considered the first priority, and nothing could have provoked a further degree of pain. The women described this “evil” as powerful enough to rob them of their “dreams,” “happiness,” “sources of support,” and “all the good memories.” Anoud described: “All my lovely dreams about getting married and being a mother have faded away.” Asera said that “cancer took away my things from me…my happiness and my husband” and Ohoud described: “The good memories had faded away.”

Taken together, these metaphors reflect the women’s shock and pain upon being diagnosed with breast cancer and many of them resonated a question: Why me?

### Battling Imminent Death

Describing the often-limited life-period of breast cancer patients, women used a range of expressions related to imminent death, as if death is around the corner: “I felt like I was going to die tomorrow” (Shatha). Fear of death metaphors included: “dying from the inside,” “my end,” and “drowning.” Women described their feelings and vulnerability in images of physical violence. Some described keeping themselves prepared for the fight against the enemy, which ongoing, as Sahara said: “Who knows? Cancer can return to start attacking me again, so I should be prepared for this!” Anoud used the expression “tearing me apart” to articulate her painful feelings when people comment about her lower chances of getting married and being a mother because of her infertility that resulted from receiving specific type of chemotherapy. In describing the pain caused by her husband’s negative words about her disease and her bodily changes, Aisha used the metaphor: “His words were sharp like knives.” The sharp knives stabbed her, threatening her life, as if it was an actual assault. Aisha also claimed that the numerous “minutes of waiting” to finish treatment were “killing” her. Having breast cancer left wounds that were hard to heal: the more the women spoke about their experiences, the more pain they described feeling, even after finishing treatment. When the first author asked Asma to share her story, Asma replied: “You are now touching my wounds and disclosing hard feelings.”

Yet, in attempting to battle death, many women used military language in verbally generating a sense of empowerment. They described themselves as “fighters,” “defendable person,” “honored,” and being involved in “ongoing war,” “battle,” “attack,” and “push.” Sahara asserted: “I prefer [to think of myself as a] fighter because it has a strong meaning—not as a hero because linked to winning. I’m still in a war against cancer, so I believe that *fighter* is the best term that describes me.” Other women expressed that they had only “beaten the cancer on the first round” and they may “lose the battle in the second-round if the cancer returned, which was highly possible.”

Impetus in fighting breast cancer spanned many motivating reasons. For example, being diagnosed with breast cancer at a young age, like Alaa, made her think about her “little daughters” who she did not want to leave behind. Other wanted to regain control over their lives and re-establish its dynamism: “To live normally” (Anoud).

These examples, amongst other aspects of the data corpus, illustrate that these Saudi women used violent, battle-theme metaphors to express their experiences. Many saw cancer as an enemy that they must defeat in order to win their lives back. Such expressions revealed confusing emotions such as being fear and vulnerable. This aligns with findings of various previous studies on the use of war–battle metaphors in describing cancer.

### Dreaming and Awakening Calls

Living with breast cancer led many women to express their changing emotions and sudden shifts in awareness like dreams, based on both pleasant and unpleasant memories, and waking up, both of which illustrated how their lives had been dramatically shaken. Both Asera and Aisha described breast-cancer treatment and its consequences as a “bad dream” from which she wishes to be “woke up.” Articulating their vulnerability were metaphorical expressions, like: “My life is filled with pain and tears” (Asera) and “The minutes of waiting are killing me” (Aisha), in which the lexical unit “minutes” is seen by Aisha as a human whose job is to kill her slowly and painfully.

Both Alaa and Leena described how they are still living the cancer experience and the persistent thoughts about cancer cannot stop, like a flashback that repeats itself. Alaa described: “It’s like a flashback that would never stop” in which she perceived the illness as a living or a moving object that will never stop chasing her. Leena said, “Before any follow-up appointment, I become very nervous and all the painful memories with cancer had returned like a flashback in front of me.” In this statement, she referred to breast cancer as painful memories which have the ability to return constantly. Shatha used a powerful metaphor that showed how her feelings constantly changed during treatment, like an elevator: “My feelings were up and down like a lift […] it was moving up and down and never stopped.”

On the other hand, many women described perceiving their experiences as positive, as a “wake-up call,” a “loving message,” and a “test from God.” Sahara described: “This disease was a blessing or a gift from God, and not a punishment.” Ohoud described paying increased attention to spiritual practices because she viewed the disease as a “sign” indicating that she should make some changes in her life. Sara shared her experience as a “break” that enabled her to spend more time with her family, reconsider her priorities, and helped her realize that life is short: “This disease is like a break that let me think about my life.”

These latter metaphors are associated with strong faith, appreciation of life, and acceptance of current situations. In these metaphors, the women described the disease as prompting them to think about themselves from different angles and find new meaning in life.

### Inner and Outer Transformation

Women used metaphors to articulate the processes of rapid change that breast cancer instigated in their bodies, as well as their emotions, which represented inescapable inner and outer transformations of their lives and identities. Words like “expired,” “marginalized,” and “broken” reflected their struggles to cope with their new selves, talking about serious and sensitive topics such as infertility. Asma mentioned that her doctor described her reproductive condition after treatment, saying, “Your ovaries are expired, like an old lady.” At this point of the interview, Asma cried because of the gravity of the symptoms not often associated with her age group; she described feeling different from other women. She then talked about her husband’s plan to get married for a second time and bring what she called “another beautiful statue to put at home.” She referred to this second wife as a beautiful object, with no rights, who could be moved or replaced at any time by the husband. Although this plan upset Asma, she justified her husband by saying that she has become “expired” or “useless” in meeting her husband’s needs.

While these metaphors revealed physical and emotional transformation in a negative sense, some women described more positive transformations. Asma and Shahad linked survivorship with the image of rescuing from drowning, which indicates awareness of their critical condition and how they were saved with good healthcare. For example, Asma described survival as “feeling a fresh oxygen filling up my lungs.” Many percipients viewed survivorship as a “rebirth” and being “born again,” suggesting new beginnings of new lives. In an exemplary metaphor, Amar summarized her experience as “thriving’ for living,” which illustrates a sense of strength and renewed motivation for life after breast cancer.

## Discussion

This study set out to assesses the use of metaphors used by Saudi Arabian women to conceptualize their experiences with breast cancer, guided by the research question: *How do Saudi Arabian women express their experiences with breast cancer using metaphors?* Four themes were generated in analysis employing MIP: Dark Hidden Force, Battling Imminent Death, Dreaming and Awakening Calls, and Inner and Outer Transformation. These themes confirm findings of previous studies and, in addition, shed light on new metaphors in making sense of the disease. Consistent with other studies that portrayed cancer as a living thing that moves, spreads and attacks in silent (Woodgate and Busolo, 2017; Leveen, 2019; Potts and Semino, 2019), many of these Saudi participants described breast cancer as “evil,” “a demon,” and “a phantom of death.” The similarities between the current study’s findings and the other related studies indicate that Saudi breast cancer patients experience similar influences and challenges as women in other contexts, and they perceive their illness experiences in similar ways. In addition, participants of this study used metaphorical expressions to reflect on their illness as an enemy, or a thief, that robs them their futures and dreams. These metaphors align breast cancer as an illness with other chronic diseases; the Saudi women described their experiences within a conceptual framework of illness metaphors in parallel with prominent themes in the literature. Some women refereed to breast cancer treatments not the disease itself as mentioned in this study, as a “violent force” that cause them fatigue, nausea, chronic pain, hair loss, and inability to do their daily life activities (Guité-Verret and Vachon, 2021). On the other hand, with the advanced in breast cancer treatment, some women perceived their illness in metaphors as a “journey” and “new normal” translating the transitional period that they went through (Appleton and Flynn, 2014; Magaña, 2020). Despite breast cancer’s relatively high survival rate, and the Western literature calls to embrace survivorship period from positive perspectives (Feuerstein, 2007; Khan et al., 2012; Cheung and Delfabbro, 2016; Park et al., 2018), some women’s metaphors illuminated the stigmatized perception of breast cancer as a death sentence.

The battle metaphors against imminent death were commonly employed in this study and similar to findings of other cancer studies (Semino et al., 2017; Magaña and Matlock, 2018; Rees, 2018). The women’s emotions moved from anger and sadness to hope and positivity, illustrating how patients’ sense of competence was both threatened and empowered by the experience of illness. Findings of this study illustrate how this disease not only threatens patients’ physical wellbeing, but their cognitive and emotional wellbeing also. The data of this study supports the claim that war metaphors in illness discourse have been mostly used to reflect negative perceptions (Harrington, 2012). However, in this study, war metaphors were also used by some participants to manifest agency and empowerment. Once many of the research participants described overcoming the shock of their diagnoses, many metaphorical narratives were directed toward their determination to fight, seeing themselves as warriors and empowered by their decisions to fight for their own lives on behalf of their loved ones. This may be partially due to the women’s cultural and religious backgrounds, where positive aspects are always intertwined with the negative, and perceptions of human existence include struggles as part of the purpose of life. As Muslims, they are encouraged to look positively toward life’s hardships and challenges as tests of their patience and faithfulness, of which they will eventually be rewarded. On the other hand, this was not the case for metastatic breast cancer patients, as it was a lost battle from their side, that reflected the medical failure in curing the disease, loss of control, and lack of shared decision-making among patients and health care providers (Guité-Verret and Vachon, 2021).

This Islamic cultural framing was also evident when women used metaphors describing breast cancer as a “wake-up call,” a “break” to think about what matters in life, and a “gift.” Theses metaphors refer to regarding their prognoses with hope and with emphasis that cancer is like a dream that inevitably has an end. Yet not every participant reflected this framing in the same way. Some described feeling stuck in a reoccurring nightmare. The instability of emotions in light of the transitional stages—which women went through feeling like they were “dreaming,” starting with breast cancer diagnosis, receiving different methods of active treatment, and dealing with short and long treatment consequences—was a very significant finding in this study. This is also evident in other studies conducted in the Middle East that identified depression, anxiety, poor body image, and fear of cancer recurrence (Fearon et al., 2020; Salem and Daher-Nashif, 2020). It is worthy to mention that what makes findings of this study unique is the fact that these Saudi women drifted away from perceiving the disease as a monster or an enemy once they started to describe a spiritual acceptance of this illness as their destiny, as Divine will, beyond their choice or control. This is in line with a recent Saudi study which found that women with breast cancer perceived their illness metaphorically in Arabic language as a “trial by ordeal” meaning that life and death are within God’s hands (Abaalalaa and Ibrahim, 2022). In these ways, the women’s Islamic cultural framing adds unique dimensions to the data, but they also provide clear evidence that conceptual meanings associated with metaphors are not fixed and stable; instead, connotations differ according to the context in which they are being used and implemented. As such, people’s language reflects both personal and social cognitions (van Dijk, 1998).

The study described how some women transformed their inner and outer perspectives, shapes, and forms during the stages of the disease. In regard to bodily changes, women expressed concerns about infertility; they complained from the lack of support of their physicians in bad news delivery, including employing such metaphors like, “Your ovaries are expired, like an old lady.” These metaphorical reflections speak to their inner voices and give insights of the challenges that young women with breast cancer face through their path of treatment and recovery. American women used certain metaphors to explain their experience with infertility as *dirty secrets* that they want to hide and struggle of being framed as *incompetent* (Palmer-Wackerly and Krieger, 2015). Adding to those findings, the current study showed that some women expressed their frustration with the way that their closest relatives, including husbands and other family members, were treating and communicating with them. Some of the women expressed concerns regards their husband’s wishes to divorce or take a second wife (Salem and Daher-Nashif, 2020). These concerns suggested that quality of life is influenced by marital relationships (Wang et al., 2019). On the other hand, some studies found that couple’s relationship became stronger after what they went through during breast cancer experience (Xu et al., 2019; Li et al., 2020).

In sum, breast cancer is a very complex disease. No one in the medical field can be perfectly accurate about patients’ prognoses and recovery. The negative effects of the treatment protocol which includes chemotherapy and radiology can be emotionally and physically damaged to both patients and their loved ones. In the case of breast cancer, like other life-threatening diseases, patients make use of metaphors to express their illness experiences in a way to reflect on their emotional and psychological cognitive perceptions (Gibbs and Franks, 2002).

### Recommendations, Limitations, and Further Research

The research participants of this study used metaphors to add vivid images to their experiences and concerns about cancer recurrence, infertility, bodily changes, desirability, caring for children, and hope for the future, which have implications at every level of society. The closest family members, including husbands, must understand what women go through with breast cancer, and psychological care is a crucial part of the overall treatment.

During communication, healthcare providers should assess the level of understanding and awareness of their patients about breast cancer, and their family members, and pay attention to the language women use as an indicator of overall wellbeing. They are encouraged to listen actively to their patients’ stories, and pay attention their choice of words, feelings, and visual images. This will strengthen the trusted relationship between healthcare providers and patients. Also, their awareness of metaphors can facilitate communication and understanding their patients, especially with the time of stress and anxiety. For example, working within Muslim cultural contexts, this study’s findings illuminated the profound role of faith and trust in God’s will, which seem to have a positive influence on women’s experience with breast cancer and resonates with the wider literature (Koenig and al Shohaib, 2014). Therefore, health care providers may benefit from spiritually framing their communication style and selecting faith-based phrases and metaphors used by Saudi Arabian women themselves. These phrases can be used by healthcare providers for the sake of patients’ acceptance their cancer diagnosis, cope with short and long consequences of treatment, and rehabilitation. Also, the inspiring key metaphors can be utilized in the visual media commercials and awareness campaigns to aid women, and their families to readjust living with and after breast cancer. Health care providers, especially who is coming from abroad, should be educated about the Saudi culture and the used common language by patients while expressing their beliefs and feelings. Also, they required to be competent in communication in order to let women feely express her thoughts and feelings.

Though, this is the first study that discuss the use of metaphors and breast cancer in the context of Arabic culture, specifically Saudi Arabia, it has several limitations. These including not addressing the effectiveness of using metaphors to cope with breast cancer. Breast cancer language was only expressed by premenopausal women Saudi Arabian women, so their experience might not reflect older breast cancer patients. Also, the generalizability is limited because the participants were young and expressed some metaphors influenced by the Islamic religion and culture and could not be applied in another different context. Since the data was collected from one health care center in the eastern side of Saudi Arabia and the sample’s homogeneity, thus, the results’ transferability into other geographical areas could not be assumed. To overcome these limitations, future research is needed to involve health care providers and family members. Recruiting participants across the country is needed to enhance the generalizability. Also, conducting quantitative and longitudinal studies would solidify the results.

There is much future research to be done. Questions exist around the degrees of metaphor intensity with differing stages of breast cancer and how patients use metaphors differently across an illness trajectory. Research is also needed on how using metaphors can affect and direct patients’ attitudes toward hope and recovery, along with cooperation with medical providers in general. Additional studies are required on the effects of medical providers’ language in their delivery of the bad news on breast cancer patients’ perceptions and implementation of metaphorical expressions in their narratives. Moreover, using metaphorical phrases to uncover hidden power relations and the understanding of social restrains is another gap in the empirical literature. Focusing on patients’ metaphorical language to analyze the correlations between metaphors pragmatic and cognitive aspects could lead to increased understanding on how emotional thinking of the world shapes the thoughts and the language women use. Moreover, future research can also examine the relationships between the language of breast cancer patients and their educational levels as well as their employment status. Findings of such studies will pave the way for brining into light other linguistic expressions associated with patients’ social backgrounds. Finally, inquiry is needed into the role of the socio-religious community and spiritual resources on breast cancer patients’ language and how their metaphorical expressions might differ according to their spiritual and religious commitment.

## Conclusion

The results of this study contribute to the growing body of research on metaphors and illness discourse; they provide better understanding of this life-threatening disease; and they offer additional evidence to the claim that metaphors are crucial to patients when communicating in relation to illness experience. They were intended to show practical implications on how a thematic analysis of metaphors in the speech of breast cancer survivors can help in providing the impact and the understanding of how research, patients and health care providers conceptualize this tough journey. Moreover, findings of this paper will increase more awareness of metaphorical language and how this can help in improving communication among patients, health care providers, and stakeholders when evaluating the process of healing and recovery. As reflected in the four themes, participants of this study employed metaphor to bring to life the bitter and sad images, resonating as speechless thoughts in their minds, and in dealing with the intensity of their cancer experiences. There is a need to increase awareness of breast cancer patients’ emotions and struggles in their inevitable journey of illness. Their voices should be heard, and their thoughts perceived, in a way that inspires their families, communities, societies, and healthcare providers to increase and continue physical, social-emotional, and spiritual support. This kind of results together with the controlled themes discussed in this study can be encouraging for future research to uncover other multi-layered conceptions of contextual linguistics in relation to chronic diseases contexts. Using metaphors to contemplate on these themes may prove effective and interesting in future health research, media communications, and other social settings.

## Data Availability Statement

The raw data supporting the conclusions of this article will be made available by the authors, without undue reservation.

## Ethics Statement

The study was approved by the Princess Nourah bint Abdulrahman University (ref 21–0257). The patients/participants provided their written informed consent to participate in this study.

## Author Contributions

WA: conceptualization. WA and MA: methodology. WA and MA: formal analysis and review and editing. Both authors contributed to the article and approved the submitted version.

## Conflict of Interest

The authors declare that the research was conducted in the absence of any commercial or financial relationships that could be construed as a potential conflict of interest.

## Publisher’s Note

All claims expressed in this article are solely those of the authors and do not necessarily represent those of their affiliated organizations, or those of the publisher, the editors and the reviewers. Any product that may be evaluated in this article, or claim that may be made by its manufacturer, is not guaranteed or endorsed by the publisher.
